# Perception of Scavengers and Occupational Health Hazards Associated with Scavenging from a Waste Dumpsite in Pretoria, South Africa

**DOI:** 10.1155/2018/9458156

**Published:** 2018-08-09

**Authors:** Senzeni Nyathi, Joshua O. Olowoyo, Agboola Oludare

**Affiliations:** ^1^Department of Biology, Sefako Makgatho Health Sciences University, P.O. Box 139, Medunsa 0204, South Africa; ^2^Department of Botany, University of Lagos, Lagos, Nigeria

## Abstract

**Objective:**

Scavenging is a source of income for most unskilled people in the developing countries. The present study investigated the perception of scavengers on scavenging and related health hazards from the Onderstepoort waste dumpsite in Pretoria, South Africa.

**Methods:**

Primary data were obtained through observation and implementation of a questionnaire to a total number of 53 scavengers (27 females and 26 males). The questionnaire was structured to extract information that included perceptions of scavengers about their activities, health implications of scavenging, monthly income, and behavioural norms.

**Results:**

Educated scavengers had high school education. The period of scavenging ranged from 10 to 15 years. The majority of scavengers did not use protective clothing. However, few used gloves and boots picked from the dumpsite. Common health issues reported included back pain, headache, diarrhoea, and shortness of breath. Some sustained injuries from sharp objects. Scavengers stored food among the waste; food could be either brought from home or bought from the vendors who cook at the dumpsite. Some drank bottled water picked from the waste. Eighty-five percent of females cleaned themselves immediately after work in temporary shacks at the dumpsite, while all males bathed at home. The average monthly income from scavenging was approximately R1372 (approximately $91 (US)). Women scavengers liked their job despite associated health risks.

**Conclusion:**

Scavengers would benefit if government and nongovernmental organisations educate them on the significance of protective clothing and good hygiene. Policymakers should assist the scavengers by providing necessary workshops on-site that will assist them to change their behaviour.

## 1. Introduction

Many people in developing countries make a living by gathering recyclable material that has been discarded by others [1], a practice called scavenging or waste picking. Valuable items are recovered from discarded solid waste [2]. The International Labour Organisation (ILO) [3] defined scavenging as physical selection of recyclable matter from muddled waste discarded at landfills, dumpsites, and places where waste is collected.

Scavengers gather discarded objects turning them into valuable substances. They change their physical form to facilitate easy transportation [1]. A study by Muhammad and Manu [4] revealed that approximately 2% of the population in developing countries make a living through scavenging. Poverty caused by unemployment in developing countries leads to scavenging [3, 5–7]. Scavenging grew due to unrestrained access to domestic bins [2]. People continue to scavenge because industrialists offer readymade market for their merchandise with the possibility of satisfying their needs [8, 9].

Gonzenbach and Coad [10] cited advantages of scavenging as working without supervision, formal dress code, and own working shifts. Scavengers may also retrieve valuables from either dustbins in residential places or dumpsites [10].

Scavenging reduces waste reaching the landfills while also providing jobs for the poor [8]. However, it is a serious health hazard. The United Nations Environmental Programme (UNEP) [11] established that scavengers may suffer from respiratory disorders due to protracted exposure to smoke from fires and dust from the waste dumpsite. Some scavengers may be injured by sharp objects resulting in death or get exposed to human immune deficiency virus (HIV) and even hepatitis infection from health care waste [12].

Magaji and Dakyes [6] established that scavengers working in Gwagwalada, Abuja, suffered from respiratory disorders, typhoid, and cholera. Scavengers may also contract skin and blood infections, eye and respiratory infections due to exposure to polluted dust, wounds and insect bites, skeletal disorder caused by the lifting of heavy storage containers, and burns due to coming into contact with hazardous chemicals combined with general waste [2, 6, 13]. Microbial load in waste dumpsites has also been found to be a serious threat to scavengers and society since scavengers carry some pathogens linked to waste into the society [13].

In South Africa, the poor are increasingly turning to scavenging despite the associated socioeconomic impact and dangers involved [1, 2, 6, 13]. This study investigated the perception and attitude of scavengers towards scavenging. The health implications associated with the practice were also highlighted. Furthermore, this study provided baseline information for policymakers in Gauteng Province on the need to have guidelines and policy on scavenging.

## 2. Materials and Methods

### 2.1. Study Area

The research was carried out at the Onderstepoort waste dumpsite in the northern part of Pretoria, Gauteng Province, South Africa. Location coordinates are approximately 25° 39′ 02″ S 28° 09′ 07″ E. This dumpsite has been operational for more than 20 years. The site is surrounded by major transport links which include the N4 route, R566, and a railway line as well as nature reserves. This dumpsite is used for domestic and industrial waste.

### 2.2. Methodology

This research used primary data obtained through observation and use of questionnaire administered to scavengers at the dumpsite. Permission to carry out the study was granted by the relevant research and ethics committees of Sefako Makgatho Health Sciences University.

On arrival at the site, permission to administer the questionnaire was sought from the site manager and the chairman of the committees formed by the scavengers. The questionnaire was administered to scavengers who were willing to participate.

A total number of 53 scavengers were interviewed. The questionnaire had different sections. The first part dealt with demography, marital status, education levels, and income, while the second part dealt with health information and risk behaviours of scavengers. The third part addressed general health information, access to health care service, and personal hygiene. The last section was developed to obtain information about the health implications of scavenging and economic benefits to the scavengers. A questionnaire was also designed to verify types of materials scavenged.

The questionnaires were administered in the morning as scavengers arrived for their day's work. Later in the day, it was difficult to get scavengers to participate since more trucks were arriving with waste, so they became busy.

## 3. Results

**Figure 1:**
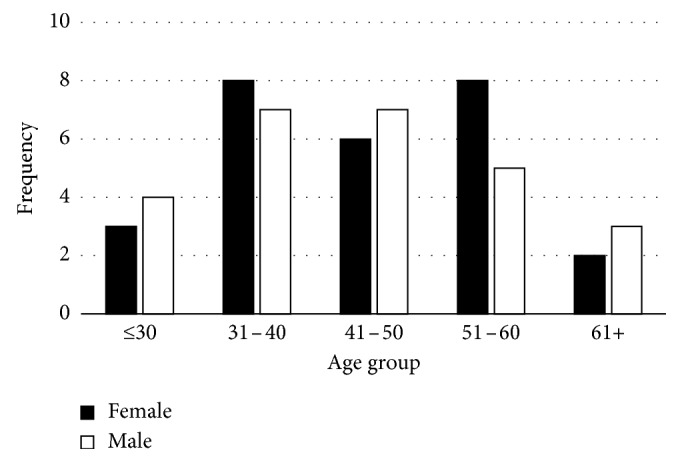
Average age distribution of the scavengers.

**Figure 2:**
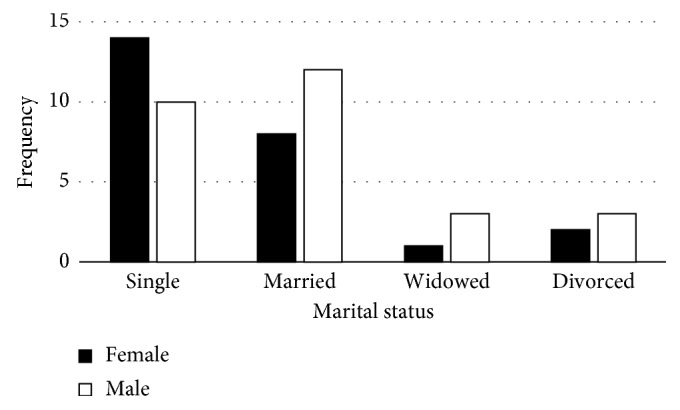
Marital status of scavengers.

**Figure 3:**
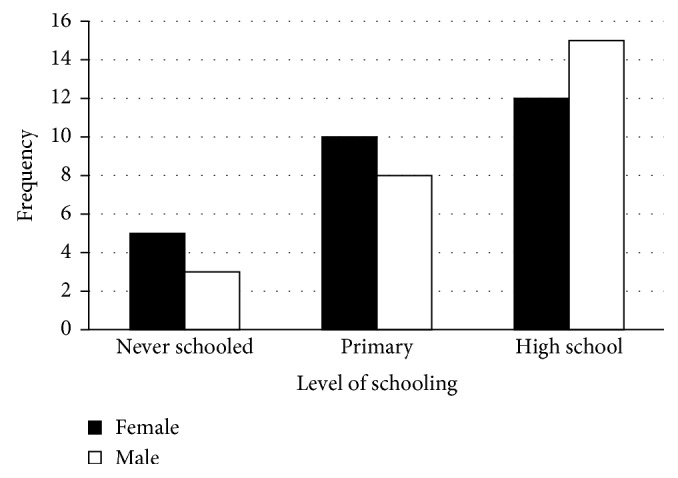
Education level of scavengers.

**Figure 4:**
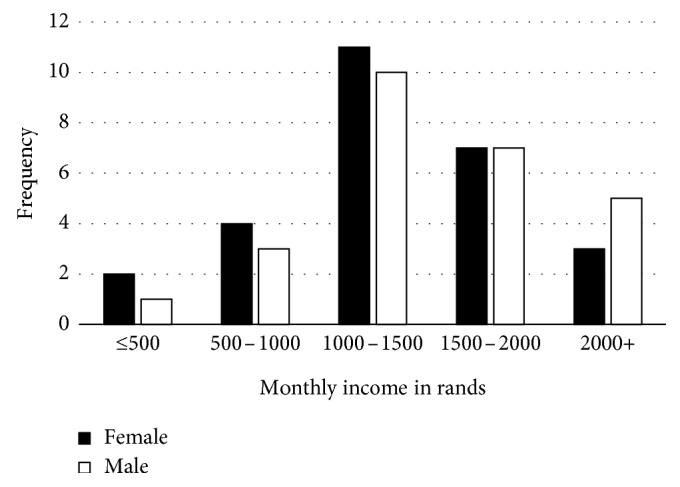
Distribution of monthly income.

The study revealed that the age of the scavengers ranged from 25 to 61 years (Figure 1), and the majority of these were single women followed by married men (Figure 2). Most scavengers went as far as high school (Figure 3). The study further revealed income bracket to be between R1000 and 1500 (Figure 4). Most of the scavengers work between 0 and 6 days per week and 6 to 12 hours per day regardless of gender. Some live in rented accommodation, a few owned houses received from the government, while others either lived with family or friends. However, 22.6% of them resided in shacks.

The study also revealed that 27.4% of females do not use self-protection attire. However, 14.8% sometimes used protection attire, and only the remaining 11.2% used protection regularly. Regarding men, 69.2% never used self-protection attire, while 11.5% of them sometimes did and 19.2% indicated that they use it regularly.

It was gathered that 44.4% of females stored food among the waste, while 38.5% of males confirmed a similar practice. One of the scavengers said “*whenever we come across left over bottled water, we do pick it for consumption*.” Scavengers do not cook at the dumpsite but buy food from food vendors cooking within the dumpsite.

Injuries were reported by 40.7% of females, while the rest said they had never been injured. Regarding men, 38.6% had been injured, while 61.5% of them said they had not been injured in the past year. Washing and cleaning of the body is performed immediately after the day's work by female scavengers in the temporary shacks using soap, and men clean themselves when they get home.

A minority of males (11.5%) have medical aid, while 88.5% rely on government hospitals. A similar situation was noticed with the female scavengers, 92.6% rely on government hospitals, and 55.5% of women do not go for annual medical checkup compared to 53.8% of males. Self-medication is practiced by 51.9% of scavengers. It was noted that 65% of men compared to 37% of women smoke. The same trend was established when it comes to the use of alcohol, 65% of men take alcohol compared to women (37%). The opposite was true for consumption of energy drinks, 51.9% of females consume energy drinks compared to 46.2% of males.

The scavenging job may constitute a serious health hazard to people who engage in this practice. In relation to wounds, 39.6% of the waste pickers admitted to having had wounds on hands and 34% had had wounds on legs. Regarding bad smell, 53% of scavengers confirmed that the bad smell from the waste affects them leading to coughing. Cutting fingernails was not a priority for scavenger, 62% of the scavengers do not regularly cut them. Only 26.9% of male scavengers wash their work garments fortnightly compared to 40.7% of females. Regardless of gender, 32% of scavengers use their garments continuously until they pick others from the dumpsite, while 34% of them have no fixed time for washing them. All 53 respondents take a bath daily. Ninety-one percent (91%) of the scavengers wash their hands with soap.

**Figure 5:**
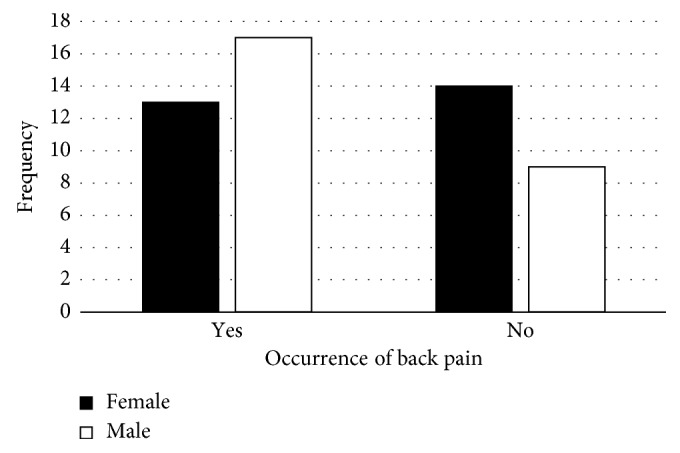
Occurrence of back pain.

Figure 5 shows that 65% of males suffer from back pain compared to 48% of females. Scavengers admitted to having symptoms such as shortness of breath (42%), headache (58%), cold/flu/cough (81%), numbness of feet (47%), conjunctivitis (24%), vomiting (34%), cramp (45%), skin rash (45%), impetigo (26%), swelling (45%), diarrhoea (38%), and feeling tired and weak (72%). 71.7% of the scavengers had seldom experienced the physical problems mentioned above.

Ninety-three percent of female scavengers said they concentrated at work without a problem compared to 89% of males. Fifty-six percent of females and 62% of males had had stress. Sleeplessness and anxiety had been experienced by 29% of females and 42% of males. Thirteen (48%) females and 65% of males felt downcast; despite this, 84% of males and 56% of females said they face problems directly. When it came to happiness, 85% of males and 63% of females professed to be happy.

**Figure 6:**
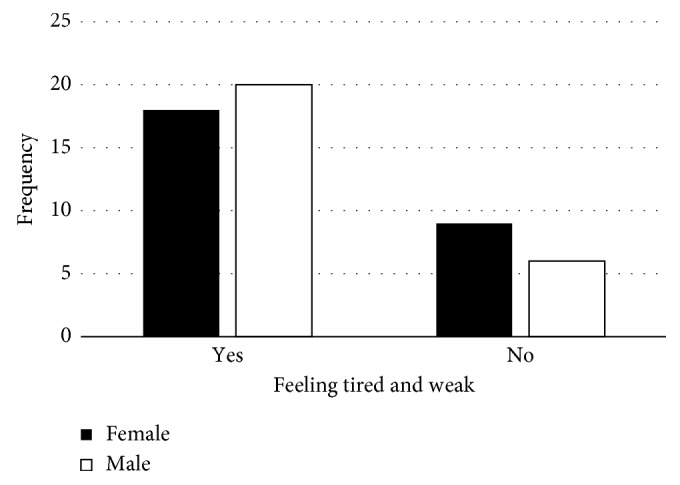
Distribution of items pick at the dumpsite.

**Figure 7:**
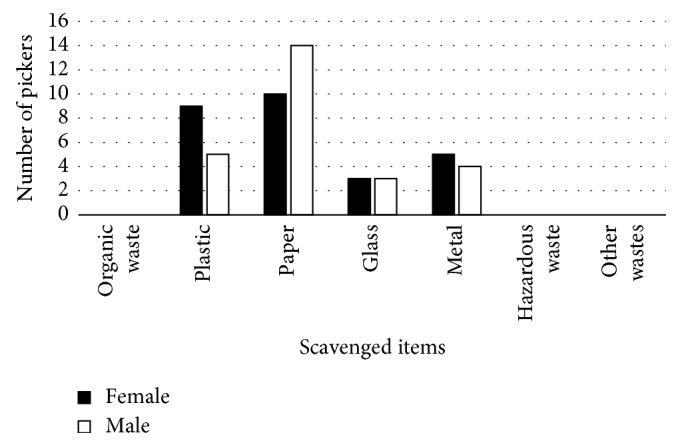
Overview of scavenger feelings.

The most scavenged items were paper, plastic, metal, and glass (Figure 6). Seventy-four percent of females and 35% of male scavengers felt the job was good, 14.8% of females and 34.6% of males felt the job was bad, and 11.1% of women and 30.7% of men were neutral (Figure 7).

Generally, 77% of the scavengers said scavenging had improved their income, while 53.5% said their health status had improved since engaging in scavenging. However, 96% of them felt that the government should legalise scavenging, and 45% confirmed that they would continue scavenging if it is legalised.

## 4. Discussion

The study established that both males and females were actively engaged in waste picking at the Onderstepoort dumpsite, in South Africa. However, this was contrary to findings by Magaji and Dakyes [6] who established that the practice was mainly conducted by males in Abuja, Nigeria. It was also concluded that there was minimal difference between the ages of females and males involved in scavenging. A further analysis of Figure 1 showed that there were more women in the age group of 51–60 compared to men in the same age group. However, the majority of female waste pickers fell within age groups of 31–40 and 51–60, while the majority of males were in the age groups 31–40 and 41–50.

This study also established that there were more single female scavengers compared to single male scavengers (Figure 2). However, a comparison between married couples showed that there were more married men compared to women (Figure 2) involved in scavenging. However, among the single waste pickers falling in the age group 31–40, the majority were females who were single parents, hence concluding that their circumstances forced them into scavenging. These findings agree with the World Bank report by Cointreau, which established that, among the 2% of the world population engaged in scavenging, the majority were women [14].

Personal interviews with individual scavengers established that the driving force behind waste picking was the scarcity of jobs. Among the common response was “*I have no alternative but to scavenge in order to put food on the table because jobs are scarce*.” However, among the scavengers, there were some who viewed scavenging as a way of raising capital to start their own business.

An analysis of the educational status of scavengers (Figure 3) showed that the highest educational standard achieved was high school. Amongst those who went as far as high school, there were more males than females. The second group of scavengers went up to primary school. In this group, there were more females than males. However, a small fraction of scavengers never went to school. This group was composed of more females than males. These findings contradict those by Simatele and Etambakonga [7], where it was established that the highest level of education attained by scavengers was primary school. These differences might be attributed to the fact that soon after independence (1994) a lot of opportunities were availed to South Africans to go to school whereas in Democratic Republic of Congo there has always been war, which hinders the majority of people from attaining high school education.

Furthermore, Simatele and Etambakonga [7] established that 36% of scavengers had no formal education, while in our study, it was established that only 15% had no formal education, an indication that the poor and the marginalised are drawn into scavenging as was previously established by Magaji and Dakyes [6].

It was further established that the income from scavenging was uniform for both men and women (Figure 4). On average, the minimum monthly income was approximately R1372. This equates to roughly $91 (US) per month and roughly $3 (US) per day. The majority of these scavengers earned below the poverty datum line. Grant [13] indicated that the lower poverty line in South Africa is R501 (about $37 (US)) per person per month, which translates to R16.50 per day (approximately $1.20 (US)).

The monthly income for scavengers was also found to be dependent on the hours spent scavenging. The more hours spent, the more income was derived. These findings were in agreement with those established by Thurarattanasunthon et al. [1] in Thailand, where it was reported that the income derived from scavenging was linked to the number of hours spent in the dumpsite. Carrasco [15] also established that the more hours spent scavenging, the more income was derived.

All scavengers working at the Onderstepoort dumpsite in Pretoria had homes to go to after work. The majority of them had families and a few lived on their own in rented rooms in the nearby townships. This was contrary to findings by Mclean [16], who established that the scavengers in Durban, South Africa, lived in the streets. Female scavengers working at Onderstepoort dumpsite were conscious about their health. They cleaned themselves just before leaving the dumpsite and also on arriving at home. On the other hand, their male counterparts only cleaned themselves when they arrived at home.

Scavengers used their clothing on average for more than a month without washing them. The majority of these scavengers apparently seemed not to understand the health risks of wearing their clothes for such long periods without washing them. Lack of hygienic knowledge among these scavengers may therefore leave them prone to bacterial infection and related pathogenic diseases. Aboadye-Larbiet al. [12] identified *Escherichia coli*, *Bacillus* sp., *Enterococcus faecalis*, and *Salmonella* sp. among bacterial microorganisms found in the dumpsites. Face-to-face interviews established that scavengers at Onderstepoort were ignorant of these microorganisms as well as the health risks they posed. This may partially explain why they took their unwashed clothing home after spending several months using them without washing. They were not aware that they were also exposing their family members and relatives to pathogens.

Additional health risk behaviour observed at Onderstepoort dumpsite was limited use of safety boots and gloves. Those that were used by a few scavengers were picked from the dumpsite. However, on the whole the scavengers did not prioritise protective clothing. These findings were similar to other studies conducted in Thailand by Thurarattanasunthon et al. [1], which established that just like in Onderstepoort in South Africa, scavengers were exposed to injuries at work due to lack of awareness on the significance of protective clothing. David et al. [17] attributed failure to prioritise protective clothing to lack of education on the part of the scavengers. This was found to be true since the majority of scavengers interviewed at Onderstepoort had not gone beyond high school and some did not have formal education.

Face-to-face interviews established that scavengers did not have a fixed time for cutting their fingernails, or bother at all about cutting them regularly. These findings confirmed the need of educating the scavengers about personal hygiene, as previously observed that most of them wore their clothes for long periods without washing them. Few if any understood that fingernails are perfect places for bacteria to flourish and that they may transfer infectious organisms into one's body. Lack of understanding of the significance of personal hygiene among the scavengers may be attributed to their low level of education since the majority of them had either high school education or only went as far as primary school (Figure 3). This situation was further aggravated by the fact that none of the scavengers received health information and that scavenging in South Africa still remains illegalised. Legalisation of the practice may pave way for nongovernmental organisations to educate the scavengers on health safety. Information on personal hygiene would also reduce ailments.

The nature of injuries proved that the main contributing factor was failure to use protective clothing. It was reported that one of the scavengers had his finger amputated after sustaining a cut by a sharp object. The majority of injuries were cuts sustained on the hands and legs. Most of the injuries could have been avoided if the scavengers wore gloves or safety boots while working. Similar injuries have since been confirmed by other scholars among the waste pickers [18, 19]. Besides injuries at the work place, the scavengers were also found to suffer from various ailments among which included low back pain, headache, conjunctivitis, vomiting, skin rash, common flu, diarrhoea, respiratory diseases, and swelling of feet. These health problems were previously reported in the World Bank report compiled by Cointreau [14]. The later also reported that scavengers suffer from respiratory illness due to ingestion of volatile substances and working in dusty conditions at dumpsites. Cointreau [14] also attributed ailments such as headaches and nausea to gases such as methane, carbon dioxide, and carbon monoxide which are found in abundance in the dumpsite.

The majority of scavengers at Onderstepoort complained of bad smell and dust which may lead to serious health problems such as chest pains. These findings concur with those by Schenckels et al. [20], who reported that breathing in dust and bad smell from the dumpsites causes chest related ailments and sinus. Despite experiencing numerous health problems, scavengers never attended annual medical checkup due to lack of medical insurance. In South Africa, the contributions demanded by medical aid schemes are perceived to be too high, hence beyond the reach of scavengers owing to the little income they derive from their business. However, the scavengers have the option to rely on state hospitals and clinics, which are funded by the state. Despite this option, it was also found that some scavengers preferred self-medication.

**Figure 8:**
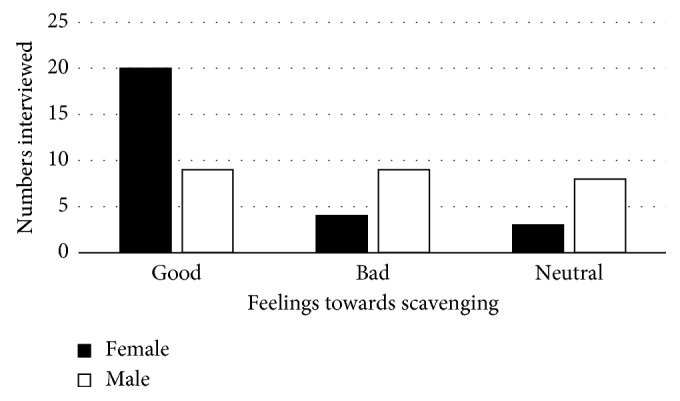
General feelings of scavengers.

None of the scavengers exhibited any drug related symptoms. Almost all the scavengers were conscious of the need of staying drug free in order to maximise profits to support themselves and their families instead of sustaining drug addiction. These findings were in contrary to those established in a similar study conducted in 2011 by Magaji and Dakyes [6] where it was reported that some scavengers used drugs for stimulation, strength, and with the view of coping with stress, abuse, and odour in the dumpsite. However, most male scavengers compared to females at Onderstepoort only consumed alcohol after a day's work or during weekends. The majority of scavengers portrayed themselves as stress free despite the majority of them admitting to feeling tired and weak whilst at work (Figure 8). However, they indicated that they continued to work hard since scavenging was their only source of livelihood. Outside their work, the scavengers lead normal lives and even participate in national elections. The small percentage which expressed reservation on participating in elections believed participation was unnecessary since it was never going to change their plight.

Face-to-face interviews further showed that more males than females experienced back pain (Figure 5). Males lifted heavier loads compared to women. In addition to handling heavy loads, the men interviewed did not understand the significance of maintaining a good posture while lifting or loading heavy goods into the trucks; hence, they were prone to suffering from back pain. Back pain problems were not only common to scavengers at Onderstepoort; Perez et al. [19] also confirmed that back pain was prevalent among scavengers. They also attributed them to heavy lifting. However, they further noted that pushing and pulling of waste containers was also another contributing factor.

The most picked item is paper followed by plastic, metal, and glass (Figure 6). According to the scavengers, these items are in demand in the recycling industry so they have a ready market. Nobody scavenges for organic and hazardous waste although some of them claimed that while scavenging they noticed used needles and dead foetuses. In South Africa, disposal of hazardous waste is regulated. It is a requirement that hazardous waste be treated first and then disposed separately in a hazardous waste landfill, which is engineered and utilised based on strict standards. According to Cointreau [14], despite strict regulations, supervision is necessary since contractors can mix the hazardous waste with nonhazardous waste in order to save money. Considering this fact, it is possible that the scavengers actually came across used needles.

Scavengers had mixed feelings about their job (Figure 7). Females seem to be satisfied compared to males. Reason given by those who claimed job satisfaction was that they determine their own hours and pace of working. Furthermore, they were not answerable to anyone. In their study, Gonzenbach and Coad [10] in 2007 postulated that the advantages of scavenging included nonsupervision, no operational regulations, coming to work at one's discretion, and no code of dressing. Men felt bad about scavenging; they do it for survival since jobs are scarce. This feeling was not peculiar to the scavengers at Onderstepoort, a study by Magaji and Dakyes [6] found that the majority of scavengers expressed their desire to leave scavenging if they had an alternative. The scavengers at Onderstepoort also indicated that if scavenging were to be legalised, they would continue working, because they are their own boss.

## 5. Limitation of the Study

Few scavengers cooperated; they feared breaching rules and regulations of the site. This resulted in few respondents as most were not sure if the study was cleared by their chairman. Others felt the study was part of police investigations on scavenging and hence were not cooperative.

## 6. Conclusion

Men and women were equally involved in scavenging, and all were poorly educated. Scavenging was found to be more acceptable among women than men. The study also revealed that many scavengers at one point suffered from back pain, cold/flu/cough, stress, diarrhoea, headache, skin rush, shortness of breath, and many other diseases associated with pollution from dumpsites. It was also found that the majority of scavengers do not use protective clothing due to economic constraints. Lack of knowledge on the dangers and health risks associated with scavenging also contributed to them being prone to illnesses.

The researchers recommend that scavenging be legalised so that the government or nongovernmental organisations can help scavengers by educating them on the importance of protective clothing and also train them on how to manage their business so that they can make profit from it.
